# Carbon monoxide poisoning: Novel magnetic resonance imaging pattern in the acute setting

**DOI:** 10.1186/1865-1380-5-30

**Published:** 2012-06-28

**Authors:** Ryan A Stephen, Sheehan W Donal, O’Neill B Siobhain, Clarkson R Michael, Costello J Daniel

**Affiliations:** 1 Department of Medicine, Neurology Service, Cork University Hospital, Cork, Ireland; 2 Department of Radiology, Cork University Hospital, Cork, Ireland; 3 Department of Medicine, Nephrology Service, Cork University Hospital, Cork, Ireland

**Keywords:** Carbon monoxide, Poisoning, Magnetic resonance imaging, Cerebellum

## Abstract

The presentation of carbon monoxide (CO) poisoning is non-specific and highly variable. The diagnosis is made when a compatible history and examination occur in a patient with elevated carboxyhaemoglobin levels. The severity of intoxication is difficult to assess accurately based on laboratory markers alone. Magnetic resonance imaging (MRI) has been shown to have superior sensitivity to computed tomography for the detection of abnormalities post CO poisoning. We report a novel imaging pattern on MRI undertaken in the acute setting in a patient with CO intoxication. We also discuss the management and follow up of patients with CO poisoning.

## Background

Carbon monoxide poisoning (CO) is common, potentially fatal, and probably under diagnosed because of its nonspecific clinical presentation. Given a typical history of CO exposure it is readily diagnosed but recognizing the condition in an unresponsive patient can be challenging. The clinical findings of CO poisoning are highly variable and largely nonspecific and laboratory markers alone offer an imperfect assessment of intoxication severity. Brain imaging is usually only undertaken if there are concerns about alternate pathology to explain neurological decompensation. However, MRI can reveal abnormalities in the deep white matter, cerebral cortex, globus pallidus, putamen, thalamus, and hippocampus. Here we report a case of carbon monoxide poisoning with an unusual pattern of bilateral restricted diffusion in the cerebellar white matter, using MRI undertaken in the acute setting.

## Case presentation

A 78-year-old lady was brought to the emergency department, having been found unconscious next to a running lawnmower. The Glasgow Coma Scale score on arrival to the emergency department was 3/15. Vital signs were recorded as a pulse rate of 78 beats per minute, temperature of 35°C, blood pressure of 110/68 mmHg and a respiratory rate of 18 breaths per minute, with oxygen saturations as assessed by pulse oximetry of 98%.

The duration of unconsciousness was unknown. Neurological examination revealed equal and reactive pupils in the midline with no focal abnormalities. The rest of the examination was unremarkable. Co-oximetry of a blood gas sample showed a carboxyhaemoglobin level of 26%. Electrocardiogram revealed no features of ischaemia and cardiac enzyme levels were normal.

She was intubated and ventilated, and an emergent CT of the brain was reported as normal. A diagnosis of encephalopathy secondary to CO poisoning was made. She was transferred to the intensive care unit and maintained on 100% oxygen. She made a prompt recovery and was extubated 8 h later. Subsequent collateral history was suggestive of a complex partial seizure disorder as the cause of her initial collapse; family members recounted multiple episodes of behavioural arrest, altered consciousness and mutism in the past.

**Figure 1:**
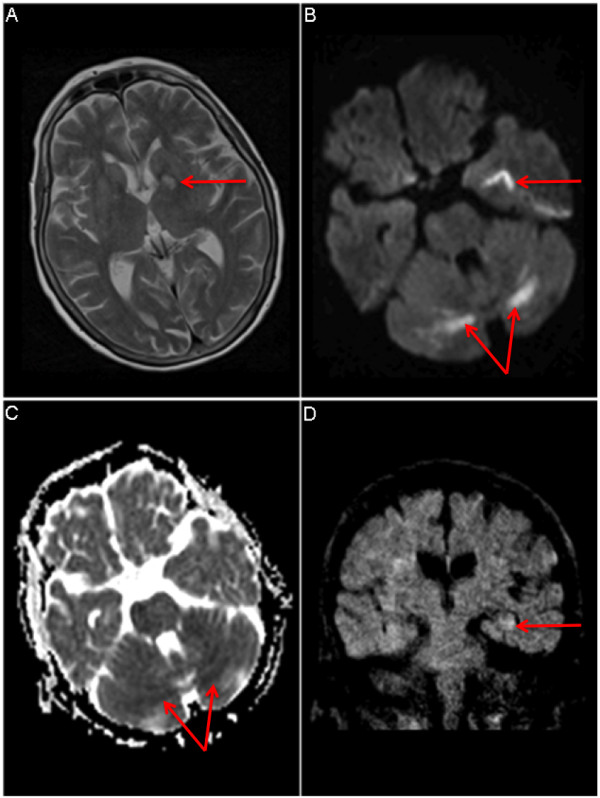
** a** T2-weighted MRI of brain with abnormal signal in the left medial temporal lobe (*arrow*). **b** Diffusion-weighted imaging (*DWI*) showing symmetrical bilateral restricted diffusion in cerebellar white matter (*arrow*) and in left medial temporal lobe (*arrow*). **c** Apparent diffusion co-efficient (*ADC*) map: corresponding areas are *dark* on ADC map, implying acute ischaemia. **d** Coronal reconstruction: Subtle signal abnormality in left hippocampus (*arrow*) possibly post-ictal in aetiology.

An MRI was performed 17 h post admission and revealed bilateral symmetrical restricted diffusion in the white matter of the cerebellar hemispheres (Figure 1). This pattern has only rarely been reported in CO poisoning and usually MRI was not undertaken in the acute setting [1,2]. Subsequent electroencephalogram (EEG) was unremarkable but importantly was undertaken 3 days post presentation. 

## Discussion

Carbon monoxide (CO) is a colourless, odourless, tasteless and non-irritant gas. Sources of CO include inadequate ventilation of appliances, faulty heating equipment and engine motors.

Symptoms of poisoning are non-specific and vary from mild constitutional upset to coma, myocardial ischaemia and death. Examination is usually unremarkable but may show subtle cognitive disturbance. Our patient presented in an unconscious state with no focal signs on examination. As the duration of unconsciousness was unknown, brain imaging was undertaken after arterial blood gas analysis confirmed CO exposure. Initial CT brain imaging was unremarkable. Magnetic resonance imaging (MRI) can reveal abnormal findings in CO-poisoned patients and is more sensitive than CT for their detection [3]. The restricted diffusion seen in our patient’s cerebellum may have been caused by ischaemia. Anatomical studies undertaken on brains of CO poisoned patients have shown abnormalities in the globus pallidus, other basal ganglia structures, hippocampus, cortex and cerebellum. These lesions are microscopically necrotic and are foci of necrosis or demyelination [4]. Cerebellar abnormalities in CO-poisoned patients are rare, and in one study that examined variables predictive of subsequent adverse outcomes, cerebellar abnormalities on presentation were associated with an increased risk of cognitive sequelae at 6 weeks [5].

Non-smokers may have up to 3% carboxyhaemoglobin whilst smokers may have levels significantly higher at 10% [6]. Standard pulse oximetry cannot differentiate between carboxyhaemoglobin and oxyhaemoglobin. The absolute level of carboxyhaemoglobin does not correlate with presence of symptoms [7], ultimate outcome [6] or white matter hyperintensities on MRI [8], but does correlate with the initial level of consciousness [6].

Carbon monoxide binds to the iron moiety of heme with approximately 240 times the affinity of oxygen [9]. Once this occurs an allosteric change results in greatly reduced off load of oxygen to tissues.

Treatment involves high flow oxygen (100% oxygen) via nonrebreathing reservoir facemask, or mechanical ventilation if indicated. This reduces the half-life of carboxyhaemoglobin from about 300 min to 90 min. Poisoned patients should be evaluated for signs of cardiovascular ischaemia and brain imaging performed if concerns of additional pathology are present. Hyperbaric oxygen therapy (HBO), which exposes patients to 100% oxygen therapy under supra-atmospheric conditions, has been shown to elevate arterial and tissue oxygen tensions, and promote CO elimination [9].

Both physiological and randomized-controlled data suggest a potential benefit [10], but uncertainty persists in identifying patients who will benefit from HBO treatment. Most authorities would administer HBO if available and the following parameters were met: severe metabolic acidosis (pH < 7.1), CO level >25, loss of consciousness or signs of end organ ischaemia.

## Conclusions

The addition of MR imaging to the diagnostic workup of CO-intoxicated patients offers additional information to clinicians to better gauge patient outcome, especially when other parameters are equivocal. Outpatient follow-up is required to assess for the development of Delayed Neuropsychiatric Syndrome (DNS), which can cause personality changes, memory difficulties and gait disturbance [11].

CO poisoning should be included in the differential diagnosis in patients found to have cerebellar white matter lesions on imaging.

## Consent

Informed consent was obtained from the patient for publication and/or presentation of this case including all medical data and accompanying images.

## Competing interests

The authors declare that they have no competing interests.

## Authors’ contributions

SR was involved in patient care, concept, drafting and critically revising the manuscript. DS was involved in drafting the manuscript and reviewing the pertinent literature. SON was involved in patient care and the preparation of the radiological aspects of the manuscript. MC personally cared for the patient and was involved in revising the manuscript. DC was involved in patient care and critically revising the manuscript for important intellectual content. All authors read and approved the final manuscript.

## Open Access

This article is distributed under the terms of the Creative Commons Attribution License which permits any use, distribution and reproduction in any medium, provided the original author(s) and source are credited.
